# Efficacy and tolerability of old and new drugs used in the treatment of immune thrombocytopenia: Results from a long-term observation in clinical practice

**DOI:** 10.1371/journal.pone.0198184

**Published:** 2018-06-01

**Authors:** Fabian Depré, Nasra Aboud, Beate Mayer, Abdulgabar Salama

**Affiliations:** 1 Institute of Transfusion Medicine, Charité Unversitätsmedizin Berlin, Berlin, Germany; 2 Department of Cardiology, Campus Benjamin Franklin, Charité Universitätsmedizin Berlin, Berlin, Germany; Centro Cardiologico Monzino, ITALY

## Abstract

**Background:**

Many patients with immune thrombocytopenia (ITP) may require special attention and long-term treatment. Little is known on the efficacy and tolerability of the drugs used in practice.

**Material and methods:**

We retrospectively reviewed the results of therapy of 400 patients with chronic ITP. All Patients were treated at our institution between 1996–2016 under consideration of guidelines, general recommendations, and individual aspects, including gender, age, weight, comorbidity, patient’s medical history and bleeding risk.

**Results:**

Treatment was not required in 25% of patients (n = 100) during observation. In treated patients (n = 300), the rate of patients that responded and tolerated treatment with prednisolone was 59% (52/88), with azathioprine 32% (29/90), with eltrombopag 49% (31/63), with romiplostim 59% 27/45, with IVIG (intravenous immunoglobulines) 75% (94/126), with anti-D 37% (19/52) and with dexamethasone 60% (25/42) patients. Eighteen treated patients (6%) entered sustained remission after treatment with various drugs. Twenty-six patients underwent splenectomy (Splx) resulting in sustained remission in 15 cases (60%). Only two patients remained refractory to Splx and to all used drugs.

**Discussion:**

None of the currently available drugs used in the treatment of ITP are invariably safe and effective. Responses, the duration of response, intolerability, and the course of disease are unpredictable. Although the treatment of ITP has considerably improved in the recent years, the currently available drugs may rarely cure affected patients. The need for safe and effective therapy in ITP is evident. Optimal treatment decisions for each patient remains a challenge in many cases.

## Introduction

Almost 100 years have passed since the establishment of splenectomy (Splx) as the first and still successfully used therapeutic measure in management of immune thrombocytopenia (ITP) [1]. The second therapeutic measure available for ITP patients was cortisone, which became available in 1951 [2], and was later on replaced by modified and less toxic steroids [3]. Since the 1960’s, immunosuppressive drugs including azathioprine, cyclophosphamide, vincristine and vinblastine have been used, but their use remains limited in ITP [4]. A new era in the treatment of ITP was established in 1981 with the observation that intravenous immunoglobulins (IVIG) resulted in an unexpected increase in platelet counts (plc) in children with ITP [5, 6]. Soon thereafter, a fourth alternative was incorporated into the list of therapeutic options in ITP: anti-D [7]. The late 1990s saw the introduction of rituximab [8], whereas the turn of the new century was influenced by well-designed studies using thrombopoietin receptor agonists (TPO-RA) [9–12]. Although the list of available drugs in the treatment of ITP is growing, there remain several unsatisfactory aspects [13]. These include the results and comments of several reviews and reports dealing with ITP management and outcomes [4, 14–22] and various guidelines on the management of ITP [23–26]. The recommendations are somewhat arbitrary and cannot be applied in many cases [14, 18, 26–29]. In addition, many factors including ethnicity [30, 31], subjective opinion, and conflict of interest may play a role in the management of ITP. Ultimately, there is no specific curative treatment for autoimmune diseases. The present study focuses on the long-term efficacy and safety of drugs used in the treatment of patients with ITP in our institute during the last two decades. The results clearly indicate that the treatment of ITP has improved, but still should be replaced by more specific and safe drugs.

## Materials and methods

Data from 400 patients (398 adults, and 2 children; 143 males and 257 females) with a mean age of 50.5 years (range, 3–101 years) that were diagnosed with chronic ITP [32], between 1964 and 2015 were retrospectively reviewed and analyzed to assess the efficacy and safety of the used therapies. All patients were treated on an outpatient basis at our institution by a single physician between 1996 and 2016. Standard dose of therapeutic agents was used (Prednisolone 0,5–1 mg/kg/d for 1–3 weeks, ≤ 7,5 mg/d for >3 weeks; Dexamethasone 40 mg/d for 4 days (1–6 cycles every 14–28 days); IVIG 0,4–2 g/kg; Anti-D 50–75 μg/kg; Rituximab 375 mg/m^2^/week for 4 times; Splx laparoscopic; Azathioprine 1-2mg/kg/d; Eltrombopag 50–75 mg/d; Romiplostim 1–10 μg/kg/week; Dapsone 75–100 mg/d; Cyclophosphamide 1–2 mg/kg/d p.o., 0,3–1 g/m^2^ i.v. every 2–4 weeks; Ciclosporine A 5 mg/kg/d for 6 days then 2,5–3 mg/kg/d (titration to 100–200 ng/ml blood level); Mycophenolate-mofetil 1000–2000 mg/d; MTX 5–25 mg/week) [22–24]. In short-course treatments (Table 1) (e.g., IVIG, dexamethasone, anti-D, rituximab, Splx), response was defined as an elevation of plc ≥ 30 x 10^9^/L with at least doubling of the baseline value within their individual agent/intervention time to peak response [32]. In patients under continuous treatments (Table 2), response criteria were a sustained elevation of plc ≥ 30 x 10^9^/L with at least doubling of the baseline value and compensated primary hemostasis during treatment observation. Sustained remission was defined as plc ≥ 100 x 10^9^/L without the requirement of further ITP therapy during at least 3 months of further observation (mean 25, months/range 3–108 months). Prior to assessment, all data has been fully anonymized. This study was approved by the institutional ethics review board (EA2/058112) of the Charité, Universitätsmedizin Berlin.

**Table 1:** Results of short-course treatments in patients with ITP during observation at our institution (n = 190).

Therapy	Patients (n)		Response			Non-R	Total ARs	Cycles in R-patients (mean/range)
	Total	Documented	Total R	Intolerability/R	SR/R			
**IVIG**	133	126	94 (75%)	-	4/94	32 (25%)	18 (14%)	3.1/1–25
**Anti-D**^**a**^	54	52	19 (37%)	-	-	33 (63%)	2 (4%)	3.8/1–19
**Dexa**	44	42	25 (60%)	1/25	-	17 (40%)	4 (10%)	1.3/1–4
**Rituxi**	4	4	1 (25%)	-	-	3 (75%)	-	1.5/1–3
**Splx**	26	25	-	-	15 (60%)	10 (40%)	1 (4%)	-

R = response (elevation of platelets ≥ 30 x10^9^/L and at least doubling of the baseline value within their known agent specific time to peak response [32]).

SR = sustained remission (sustained platelet count ≥100 x 10^9^/L without any further ITP therapy during at least 3 months of further observation).

Non-R = non-responder (patients that did not show elevation of platelets ≥ 30 x10^9^/L and at least doubling of the baseline value)

ARs = adverse reactions

Documented = patients with available follow-up data

Dexa = dexamethasone, IVIG = intravenous immunoglobuline, Rituxi = rituximab, Splx = splenectomy

^a^ One patient with Splx prior to anti-D treatment

**Table 2:** Results of continuous treatments in patients with ITP during observation at our institution (n = 234).

Therapy	Patients (n)		Response			Non-R	Total ARs	Treatment time in months (mean/range)
	Total	Documented	Total R	Intolerability/R	SR/R			
**Pred**	92	88	64 (73%)	12/64	8/64	24 (27%)	30 (34%)	21.2 / 1–210
**Aza**	14	12	7 (58%)	1/7	1/7	5 (42%)	4 (33%)	13.6/0.5–48
**Aza + Pred**	82	78	42 (54%)	19/42	1/42	36 (46%)	34 (44%)	15/0.5–108
**Romip (+Pred**^**a**^**)**	52	45	35 (78%)	8/35	-	10 (22%)	15 (33%)	12,1/0.75–72
**Eltromb**	64	63	45 (71%)	14/45	4/45	18 (29%)	22 (35%)	15.6/0.5–96
**MTX (+Pred**^**b**^**)**	5	5	2 (40%)	-	-	3 (60%)	-	48.2/2–175
**Cycl (+Pred**^**c**^**)**	6	6	4 (66%)	-	-	2 (33%)	2 (33%)	13.3/1.5–29
**Ciclo (+Pred**^**d**^**)**	12	10	1 (10%)	-	-	9 (90%)	1 (10%)	5.8/0.75–18
**MMF (+Pred**^**e**^**)**	15	14	7 (50%)	3/7	-	7 (50%)	6 (43%)	15.7/ 1–60
**Dapsone**	3	3	1 (33%)	-	-	2 (66%)	-	41.3/ 2–120

R = response (sustained platelet counts ≥30 x10^9^/L with at least doubling the baseline value and absence of bleeding during observation).

SR = sustained remission (sustained platelet counts ≥100 x 10^9^/L without any further ITP therapy during at least 3 months of further observation).

Non-R = non-responder (patients that did not show elevation of platelets ≥ 30 x10^9^/L and at least doubling of the baseline value).

ARs = adverse reactions

Documented = patients with available follow-up data

Aza = azathioprine, Ciclo = ciclosporine, Cycl = cyclophosphamide, Eltromb = eltrombopag, MMF = mycophenolate- mofetil, MTX = methotrexate, Pred = prednisolone, Romip = romiplostim

^a^ Five patients with concomitant prednisolone therapy;

^b^ four patients with concomitant prednisolone therapy;

^c^ five patients with concomitant prednisolone therapy;

^d^ one patient with concomitant prednisolone therapy;

^e^ twelve patients with concomitant prednisolone therapy

## Results

**Fig 1:**
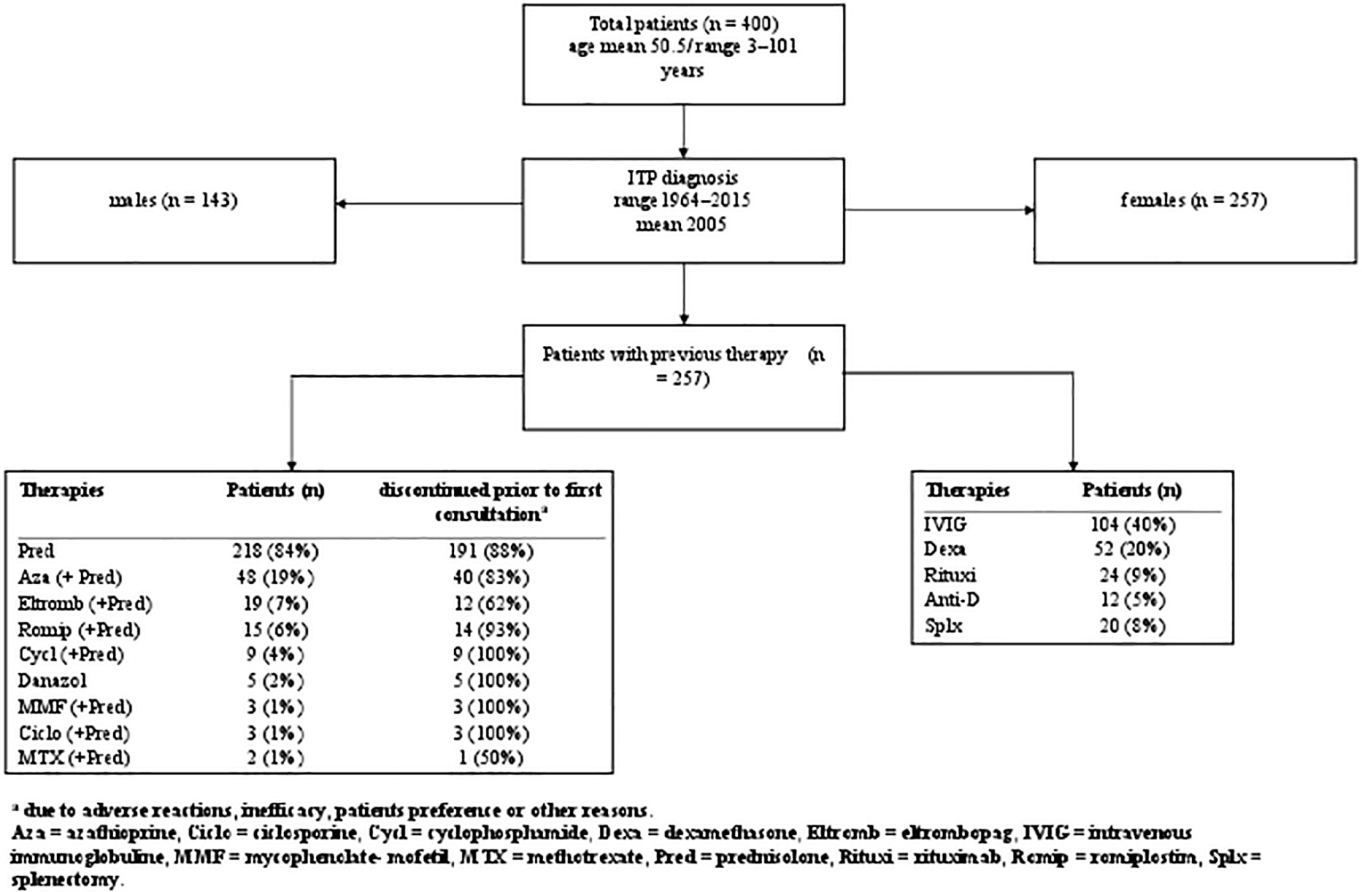
Patient and clinical characteristics.

Prior to consultation at our institution, 257 of 400 patients underwent treatment with therapies that were ineffective and/or intolerable due to side effects (Fig 1). The mean number of previous therapies was two (range 1–6). Prednisolone was the most frequent previously used drug (84%). The vast majority of these patients (88%) discontinued prednisolone due to inefficacy and/or intolerability including weight gain, hypertension, diabetes, osteoporosis, gastric ulceration or depression, resulting in a switch in therapy prior to initial consultation at our institution (Fig 1).

**Fig 2:**
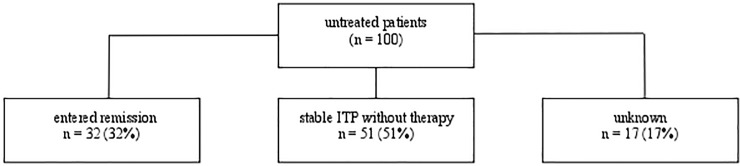
Patients that just required controls due to mild ITP without any therapy during observation.

One hundred (25%), including the two children, of the 400 patients did not require any therapeutic intervention during observation due to mild ITP and the absence of any bleeding and/or high bleeding risks. Of these 100 patients, 32 (39%) entered remission, 51 patients (61%) had persistent, but still mild ITP without the need for therapy, and long-term follow-up was absent in 17 patients (Fig 2).

The remaining 300 patients received an average of two treatments (range 1–10) during observation at our institute.

A total of 92 patients (23%) received prednisolone for at least one month (Table 2). Follow-up data were not available in four cases (4%). In the remaining 88 patients, 64 (73%) responded to this treatment. However, only eight (13%) of the responding patients entered sustained remission. Despite low-dose therapy (1–7.5 mg/d; Table 2), 12 (19%) patients developed intolerability during observation. Twenty-four patients (27%) did not respond to treatment.

Azathioprine was used in 96 patients (24%), 82 of those had additional concomitant treatment with low-dose prednisolone (1–7.5 mg/d; Table 2). Six patients (6%) were not evaluated due to lack of follow-up data. In the remaining 90 patients, 49 patients (54%) responded to the treatment and 20 (41%) of those patients discontinued therapy due to adverse reactions, which included diverse gastrointestinal symptoms, hair loss, and drug fever. Two patients (2%) entered sustained remission without the need for further ITP therapy. Forty-one patients (46%) did not respond to the drug.

Forty-four patients (11%) were treated with dexamethasone at least once during observation (Table 1). Two patients (5%) were not evaluated due to lack of follow-up data. Of the remaining 42 patients, 25 (60%) responded to treatment (Table 1). Adverse reactions (headache, insomnia, mood disturbances, agitation, anxiety) were observed irregularly, and were tolerable in most cases. Seventeen patients (40%) did not respond to dexamethasone.

Other immunosuppressive drugs including, cyclophosphamide, ciclosporin, mycophenolate- mofetil and rituximab showed response rates ranging from 10 to 66% (Tables 1 and 2). In addition, combination therapy with the aforementioned drugs and concomitant prednisolone therapy (≤ 7,5 mg/d) was used in some patients (Table 2). Adverse reactions observed during treatment with immunosuppressive drugs were largely in agreement with previously reported data [22–25].

Fifty-two patients (13%) were treated with romiplostim (Table 2). Seven patients (13%) were not evaluated due to lack of follow-up data. Of the remaining 45 patients, 35 patients (78%) responded to the drug. Treatment was discontinued in 8 (23%) of the responding patients due to adverse reactions [33, 34]. None of the patients treated with romiplostim entered sustained remission. A total of 10 patients (22%) did not respond to romiplostim. Eltrombopag was used in 64 patients (16%; Table 1). One patient (2%) was not evaluated due to lack of follow-up data. Of the remaining 63 patients, 45 patients (71%) responded to treatment (Table 2). Four of these patients (9%) entered sustained remission. Fourteen (31%) of the 45 responding patients discontinued treatment due to intolerable adverse reactions [33, 34]. Eighteen patients (29%) did not rspond to eltrombopag.

IVIG was used in 133 patients (33%) on at least one occasion (Table 1). Seven patients (5%) were not evaluated due to lack of follow-up data. Of the remaining 126 patients, 94 (75%) responded to treatment, and none of the responding patients developed intolerable adverse reactions that contraindicated a re-treatment with IVIG. The observed adverse reactions were usually mild and related to headaches and rarely allergic reactions. Interestingly, four patients (4%) entered sustained remission following IVIG treatment (Table 1). Thirty-two patients (25%) did not respond to IVIG.

Therapy with anti-D was selectively used in 54 Rh(D)-positive patients (14%) with refractory ITP (Table 1). Two patients (4%) were not evaluated due to lack of follow-up data. Nevertheless, of the remaining 52 patients, 19 (37%) responded to anti-D and two patients (4%) had adverse reactions (nausea, fever, headache) 24 h after administration that may be attributed to other origins. Hemolysis was not observed in a single patient. The lack of hemolysis might be explained by the subcutaneous administration [35–38]. Thirty-three patients (63%) did not respond to anti-D.

Splx was performed in 20 patients (5%) prior to consultation at our institute. Of the remaining 380 non-splenectomized patients, 26 (7%) underwent Splx during the observation period (Table 1). One patient (1%) was not evaluated due to lack of follow-up data. Of the remaining 25 patients, 15 (60%) patients entered sustained remission after Splx. One non-responding patient developed postoperative abdominal pain and was diagnosed portal vein thrombosis (Table 1).

## Discussion

The present study mainly focused on the efficacy and tolerability of each drug used in practice in the treatment of ITP rather than on patient outcome. While the latter question has been acknowledged by several studies [39–42], the former question has not yet been adequately addressed. The results presented in this study are based on patients treated by one physician who treats ITP patients since the 1980, not only on the basis of general recommendations, but also under individual consideration of each patient’s conditions. These included prior therapy, bleeding risk, plc, gender, age, weight, comorbidity, quality of life and patient’s preference. The importance of the latter point is reflected by the fact that many patients are well-informed on ITP. Based on the aforementioned criteria, at least 25% of patients did not require any treatment during observation (Fig 2). These patients did not have any significant bleeding or bleeding risk due to comorbidities, menstruation, activities/sports associated to injuries or invasive medical interventions. Their plc remained at levels compensating primary hemostasis (≥ 30 x 10^9^/L) during observation. This observation is in agreement with previously published data [23].

Drug-induced thrombocytopenia rather than ITP was suspected in 24 patients. At least nine of these patients (38%) entered sustained remission following discontinuation of the suspected drug.

Unfortunately, many patients (55%) received steroids prior to consultation at our institute, even in cases where steroids should have been avoided, e.g., in patients with obesity, diabetes, and/or hypertension. Based on the aforementioned criteria, treatment with steroids was preferred as first-line therapy in only 31 of 143 patients that had not been treated for ITP prior to admission in our institute. Of these selected patients, 20 (65%) showed long-term beneficial effects that may have been solely attributed to steroids. However, these patients were a selected group of uncomplicated cases, e.g. young individuals with few or no comorbidities, with mild rather than severe ITP, no recognizable contraindication for treatment with steroids, and appeared to have a relatively low risk of developing intolerance. As the majority of such selected patients appeared to respond to steroids, we believe that this supports the notion of retaining steroids as a first-line therapy in ITP, however only in selected patients. In comparison, only (12%) of pretreated patients (27/257), still remained under steroid treatment upon initial presentation at our institute (Fig 1), and this treatment was discontinued in six of these cases during observation. Therefor the number of patients that continued treatment (10%) is primarily in agreement with previous reports [4].

Anti-D was selectively used in 54 Rh(D)-positive patients with severe ITP that were largely refractory to previous medical therapy (Table 1). This may explain the relatively low response rate (37%) compared to unselected patient cohorts [4]. The reason for anti-D being not used in uncomplicated (naïve) patients is related to the fact that this therapeutic option is still not licensed for treatment of ITP in Germany.

Prior to admission, 20 patients (5%) of the patient cohort were already splenectomized and four of them did not require any further medication. The other 16 patients required further therapy. During observation, only 26 (11%) of the remaining 284 non splenectomized and treated patients remained refractory to all available drugs, and ultimately underwent Splx. Fifteen of these patients (60%) entered sustained remission, and 10 (40%) required further treatments (Table 1). Of these 10 patients, seven patients responded to treatment with TPO-RA (six patients to romiplostim, one patient to eltrombopag), and the remaining patient responded to azathioprine and prednisolone (≤ 7,5 mg/d). Altogether, the therapies used in 26 splenectomized patients who required further therapy, were more effective than that used prior to Splx (Table 3).

**Table 3:** Therapies prior to and after splenectomy.

Total number of splenectomized patients (n = 46)		Patients receiving treatment after Splx (n = 26)			
Abortive therapies prior to Splx	Patients (n)	Therapies after Splx	Patients (n)	Response	Non-R
**Pred**	42	**Prednisolone**	5	3 (60%)	2 (40%)
**IVIG**	28	**IVIG**	12	9 (75%)	3 (25%)
**Dexa**	9	**Dexa**	2	1 (50%)	1 (50%)
**Anti-D**	12	**Anti-D**	1	-	1 (100%)
**Romip**	6	**Romip**	13	10 (77%)	3 (33%)
**Eltromb**	7	**Eltromb**	8	4 (50%)	4 (50%)
**Rituxi**	4	**Rituxi**	2	-	2 (100%)
**Aza + Pred**	5	**Aza + Pred**	6	2 (33%)	6 (66%)
**Aza**	4	**Aza**	1	1 (100%)	-
**Cycl**	5	**Cycl (+Pred**^**a**^**)**	2	1 (50%)	1 (50%)
**Ciclo**	3	**Ciclo**	1	1 (50%)	-
**MMF**	4	**MMF (+Pred**^**b**^**)**	2	-	2 (100%)
**Dapsone**	1	**Dapsone**	-	-	-

Response = short-course treatments: elevation of platelets ≥ 30 x10^9^/L and at least doubling of the baseline value within their known agent specific time to peak response [32]. Continuous treatments: sustained platelet counts ≥30 x10^9^/L with at least doubling the baseline value and absence of bleeding during observation.

Non-R = non-responder (patients that did not show elevation of platelets ≥ 30 x10^9^/L and at least doubling of the baseline value).

Aza = azathioprine, Ciclo = ciclosporin, Cycl = cyclophosphamide, Dexa = dexamethasone, Eltromb = eltrombopag, IVIG = intravenous immunoglobulin, MMF = mycophenolate- mofetil, MTX = methotrexate, Pred = prednisolone, Romip = romiplostim, Rituxi = rituximab, Splx = splenectomy.

^a^ One patient with concomitant prednisolone therapy;

^b^ one patient with concomitant prednisolone therapy.

The finding that most therapeutic options were observed to become effective after Splx continues to emphasize the role of this therapeutic measure in the management of ITP as long as there is no specific treatment available. A similar conclusion has been drawn in previous studies [43, 44]. Most intriguingly, the efficacy of splenectomy appears to be independent of previous therapies and may therefore be considered as the ultima ratio therapy in refractory patients. Only two of the treated patients in this study remained refractory to all drugs used, even after splenectomy. One of these, a 53 year old female, who remained refractory to all drugs used (high-dose IVIG, platelet transfusion, eltrombopag, romiplostim, dexamethasone, dapsone and ultimately myeloablative dose cyclophosphamide 4 x 50 mg/kg/d) post-splenectomy. At first admission her blood count revealed only isolated thrombocytopenia and the blood marrow smear and bone marrow aspiration showed no abnormalities. This patient developed acute myeloid leukemia and died several weeks following treatment. The second refractory patient remained at risk of bleeding.

The results obtained using immunosuppressive agents were similar to those reported in previous studies [4]. Interestingly, similar response rates were observed in patients treated with azathioprine and treated with both azathioprine and low-dose (1–7.5 mg/d) prednisolone (50% *vs*. 51%). Other immunosuppressive drugs including cyclophosphamide, ciclosporin and mycophenolate-mofetil were only used in refractory patients. In most cases, the combination treatment of steroids and other immunosuppressive drugs was required to reduce steroid doses. A small group of 5 patients suffering from rheumatic diseases (3 patients with rheumatoid arthritis, 1 patient with systemic lupus erythematodes, 1 patient with psoriasis arthritis) was treated with MTX. None of these patients has been treated with MTX because of ITP. Nevertheless, a therapeutic effect of MTX on ITP, cannot be excluded. Two patients with concomitant prednisolone therapy met the response criteria during this therapy (Table 2).

IVIG was administered to 133 patients, with 94 patients (75%) responding to treatment (Table 3). Thirty-two patients (25%) did not show an increase in plc as defined by the response criteria. In patients with bleeding, bleeding did not progress or was stopped after IVIG administration. We recently demonstrated that high dose IVIG may stop bleeding within 12 h after administration, independent of plc [45]. This phenomenon might be explained by an increased consumption of platelets for normalization of decompensated primary hemostasis in treated patients.

The TPO-RA, romiplostim and eltrombopag are increasingly used in the treatment of ITP. Previous studies have shown both drugs to be generally effective and safe in most ITP patients [9–12, 33, 34, 46–48]. However, in some cases, severe or dangerous adverse reactions including thromboembolic events, arthralgia, increased bone marrow fibrosis and myeloproliferative neoplasias may occur [9–12, 33, 34, 46–48]. Our results indicate that TPO-RAs are less effective and less tolerated (49–59%) than previously reported and may potentially result in an increased number of adverse reactions than previously reported [9–12, 33, 34, 46–48]. Adverse reactions occurred in 33% (romiplostim) and 35% (eltrombopag) of patients, respectively. Of the responding patients, eight (23%) discontinued romiplostim, and 14 (31%) discontinued eltrombopag due to intolerable adverse reactions. Nevertheless, these drugs have not only changed therapeutic treatment options in ITP, but also the outcome of treated patients. Previous studies have shown that approximately one-third of patients remained refractory to conventional treatment [49]. Currently, approximately <10% of patients may remain refractory to treatment [50, 51] and death may occur only in isolated cases [52]. In addition, the use of immunosuppressive drugs can nowadays rarely be justified.

Finally, independent of Splx, 18 patients (6%) entered sustained remission (plc ≥100 x 10^9^/L) due to treatment with various drugs (Tables 1 and 2). It remains unknown whether this phenomenon can only be attributed to the applied drugs mentioned in this study.

## Conclusions

Although only isolated patients may remain refractory to the currently available treatment options in ITP, there are many aspects that remain evident for the need of alternative drugs including specificity, safety, efficacy, predictability, and costs. In addition, we and others [13, 14, 27] agree with guidelines as a source of basic information, but individualized treatment is needed in many cases.

### Limitations of study

As the Dataset, collected retrospectively, does not contain uniquely all parameters for each patient, we prefered to conduct only descriptive instead of inferential statistics. Our retrospective study represents the treatment results in clinical practice. Thus, there was no possibility of monitoring platelet counts at prespecified standardized moments as in prospective trials. Moreover, the time to response is variable and cannot be predicted. It must be emphasized that not all adverse reactions were excluded, e.g. bone marrow fibrosis. In addition, a systemic screening for portal vein thrombosis by ultrasound or CT-scan after Splx was not performed. Furthermore our study presents data collected over a period of 20 years. During this period, therapeutic options as well as treatment recommendations have changed.

Initially, most patients appeared to have isolated ITP. During observation, at least 57% (n = 227) of our patients exhibited other abnormalities e.g., DAT positivity, antibody deficiency, malignancies, or other diseases and abnormalities, Based on our experiences [53], the common classification into a primary and a secondary disease is not certain and variable in many cases. Moreover, the ITP itself might be responsible for the development of associated diseases at least in some cases. This is confirmed by the matter of fact that the manifestation of ITP precedes the manifestation of the associated diseases in some patients. Thus the question which disease is the primary one remains vague.

## Supporting information

S1 File Dataset. (XLSX)
